# State of non-communicable diseases in Nepal

**DOI:** 10.1186/1471-2458-14-23

**Published:** 2014-01-10

**Authors:** Gajananda Prakash Bhandari, Mirak Raj Angdembe, Meghnath Dhimal, Sushma Neupane, Choplal Bhusal

**Affiliations:** 1 Nepal Public Health Foundation, 102/2 Dhara Marg, Maharajgunj, P.O. Box: 11218, Kathmandu-4, Nepal; 2 Department of Public Health, Central Institute of Science and Technology, Pokhara University, Kathmandu, Nepal; 3 Nepal Health Research Council, Kathmandu, Nepal

**Keywords:** Non-communicable disease, Nepal, Prevalence, Chronic disease, Burden, Magnitude

## Abstract

**Background:**

The prevalence of Non Communicable Diseases (NCDs) is still unknown in Nepal. The Ministry of Health and Population, Government of Nepal has not yet formulated policy regarding NCDs in the absence of evidence based finding. The study aims to find out the hospital based prevalence of NCDs in Nepal, thus directing the concerned authorities at policy level.

**Methods:**

A cross sectional study was conducted to identify the hospital based prevalence of 4 NCDs (cancer, cardiovascular disease, diabetes mellitus and chronic obstructive pulmonary disease), wherein 400 indoor patients admitted during 2009 were randomly selected from each of the 31 selected health institutions which included all non-specialist tertiary level hospitals outside the Kathmandu valley (n = 25), all specialist tertiary level hospitals in the country (n = 3) and 3 non-specialist tertiary level hospitals inside the Kathmandu valley. In case of Kathmandu valley, 3 non-specialist health institutions- one central hospital, one medical college and one private hospital were randomly selected. The main analyses are based on the 28 non-specialist hospitals. Univariate (frequency and percentage) and bivariate (cross-tabulation) analysis were used.

**Results:**

In non-specialist institutions, the hospital based NCD prevalence was 31%. Chronic obstructive pulmonary disease (43%) was the most common NCD followed by cardiovascular disease (40%), diabetes mellitus (12%) and cancer (5%). Ovarian (14%), stomach (14%) and lung cancer (10%) were the main cancers accounting for 38% of distribution. Majority of CVD cases were hypertension (47%) followed by cerebrovascular accident (16%), congestive cardiac failure (11%), ischemic heart disease (7%), rheumatic heart disease (5%) and myocardial infarction (2%). CVD was common in younger age groups while COPD in older age groups. Majority among males (42%) and females (45%) were suffering from COPD.

**Conclusions:**

The study was able to reveal that Nepal is also facing the surging burden of NCDs similar to other developing nations in South East Asia. Furthermore, the study has provided a background data on NCDs in Nepal which should prove useful for the concerned organizations to focus and contribute towards the prevention, control and reduction of NCD burden and its risk factors.

## Background

Non-communicable diseases (NCDs) refer to diseases or conditions that occur in, or are known to affect individuals over an extensive period of time and for which there are no known causative agents that are transmitted from one affected individual to another [1]. The risk factors for many of the NCDs are associated with lifestyle related choices, environmental and genetic factors. Tobacco use, harmful use of alcohol, unhealthy diets (high in salt, sugar and fat and low in fruits and vegetables) and physical inactivity are some of the established behavioral risk factors of NCDs.

A large proportion of global morbidity and mortality could be attributed to NCDs. It was estimated that globally in 2008, cardiovascular diseases or CVDs (17 million deaths, or 48% of NCD deaths), cancers (7.6 million, or 21% of NCD deaths), respiratory diseases, including asthma and chronic obstructive pulmonary disease (COPD) (4.2 million) and diabetes (1.3 million deaths) were primarily responsible for staggering 36 million deaths (63% of global deaths) [2]. Traditionally regarded as a woe of the high income countries, NCDs are becoming increasingly prevalent in low and middle income countries with the emergence of lifestyle changes. NCDs are turning out to be the principal causes of deaths in these countries where an estimated 80% of NCD deaths occurred [2].

In low to high income countries 50-80% of health budget is spent on chronic diseases [3]. This is particularly a larger problem in low-income settings, where double burden of infectious as well as chronic diseases are straining their health services [4,5]. South Asia is one of the populous as well as the poorest regions of the world where NCDs account for nearly 50% of disease burden in adult population [6]. In this region in 1998, the proportion of deaths due to NCDs ranged from 7% in Nepal to 48% in Sri Lanka [7].

In Nepal, prevalence of coronary heart diseases in eastern region was 5.7% in 2005. Similarly prevalence of hypertension was 22.7% in Dharan municipality [8]. Studies have shown that the prevalence of hypertension in adult population was around 20% in urban population [9]. According to the data of 'Sunsari Health Survey’ of the year 1993, the prevalence of diabetes and hypertension in Sunsari District, from eastern Nepal, was about 6% and 5.1% respectively in adults [10]. A more recent data from an urban area has shown the prevalence of diabetes and impaired fasting glucose as 14.2% and 9.1% respectively [11]. Different studies report different prevalence of diabetes in different age groups and sites. Studies show prevalence of 19% among people aged 40 plus years [12] and 25.9% in elderly aged 60 years and above [13].

Majority of the NCDs could be easily avoided through life style changes like eating a healthy diet, avoiding tobacco use and exercising regularly. These are simple cost effective interventions that have worked in different settings around the world [14]. Such interventions alone could lead to a reduction of 80% heart disease, stroke, and type 2 diabetes, and 40% cancer [14]. However NCDs still remain a neglected issue in the agendas of most of the donors and governments around the world [15].

Nepal is one of the poorest countries in the world - at 157th position of Human Development Index [16]. The prevalence of NCDs is still unknown. The Ministry of Health and Population, Government of Nepal has not yet formulated policy regarding NCDs in the absence of evidence based finding. Thus it is important to address the burden of NCDs through research.

For this purpose initially hospital based prevalence data generated from the regional, sub-regional, zonal and specialist centers across the country was targeted. This study was expected to provide a baseline data on prevalence of NCDs (cancer, CVD, diabetes mellitus and COPD) in Nepal. In particular, it aims to find out the hospital based prevalence of NCDs in Nepal, thus directing the concerned authorities at policy level.

## Methods

This was a cross sectional study to identify the hospital based prevalence of 4 NCDs (cancer, CVD, diabetes mellitus and COPD). Thirty one health institutions (central, regional, sub-regional, zonal hospitals, medical colleges and specialist centers) were selected from the five development regions. In Nepal, most of the cases of NCDs are treated in the tertiary level health institutions like central, regional, sub-regional, zonal, specialist hospitals and medical colleges. District level hospitals have few facilities for the diagnosis and treatment of NCD cases so they refer these cases to tertiary level health institutions. Taking these issues into account, we selected all non-specialist tertiary level hospitals outside the Kathmandu valley (n = 25), all specialist tertiary level hospitals in the country (n = 3) and 3 non specialist tertiary level hospitals inside the Kathmandu valley to calculate the hospital based prevalence of NCDs. In the case of Kathmandu valley, since there are many non-specialist tertiary health institutions, 3 were randomly selected- one central hospital, one medical college and one private hospital. For completeness, all three specialist centers (Bhaktapur Cancer Hospital, B.P. Koirala Memorial Cancer Hospital and Sahid Gangalal National Heart Center) in Nepal were also selected.

This study was conducted over a period of eight months from December 2009 to July 2010. Indoor patients, 35 years or older were included in the study. This study included only the indoor patients of the selected hospitals because hospitals maintain detailed case records of indoor patients only and they were easily accessible for the study.

Sample size was estimated on the basis of unknown prevalence of NCD (50% assumed for conservative sample size estimates) with 5% allowable error and 95% confidence level. The sample size calculated was 384. This figure was rounded so that 400 cases were randomly selected from each selected health institution. During this process, the hospital records were reviewed to obtain the information regarding the total number of indoor cases registered in the year 2009. Then cases were selected using computer generated random numbers (Ms-Excel 2007) until the required sample size of 400 was reached. In this study, cases were patients diagnosed by physician as cancer, COPD, diabetes mellitus (DM) or CVD (as stated in the records) and who were admitted in indoor wards of selected health institution. If the selected case did not fulfill the inclusion criteria, then immediate next number was taken as a case. Details like inpatient number, age, sex, ethnicity, address and diagnosis were then obtained. Checklist and data compilation forms were used for this purpose. Enumerators were hired and trained for data collection. They visited each of the selected health institutions and carried out their task.

Data obtained were coded and entered in Ms-Excel 2007. The data base was then exported to SPSS (ver. 11.5) for analysis. Univariate analysis was carried out using frequencies and percentages. In bivariate analysis cross-tabulations were used. The study was approved by the ethical review board of Nepal Health Research Council. Formal permission was obtained from the concerned authorities of the selected health institutions. Confidentiality was maintained.

## Results

This study was conducted in 31 health institutions (central, regional, sub-regional, zonal hospitals, medical colleges and specialist centers) of Nepal for the purpose of identifying the hospital based prevalence of NCDs. The total number of patients admitted to these hospitals in 2009 was 347,261; out of which 11,901 cases were randomly selected. The number of cases selected from each of the health institutions ranged from 350–400.

### Proportion of NCDs in non-specialist institutions

**Figure 1:**
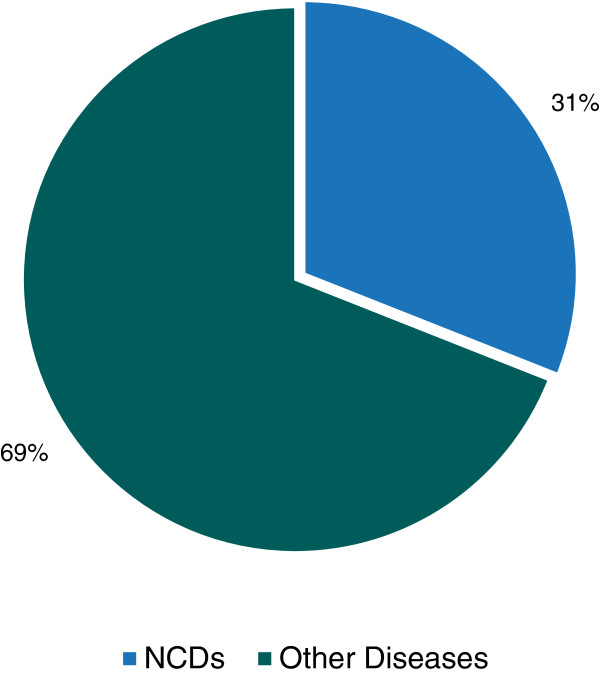
Proportion of NCDs in non-specialist institutions, 2009.

**Figure 2:**
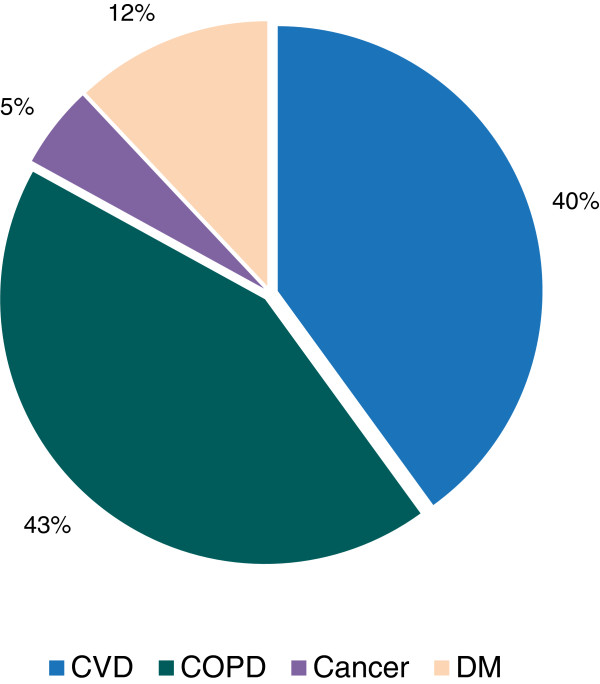
Proportion of various NCDs in non-specialist institutions, 2009.

NCDs accounted for 31% (3,294) of all admitted cases in 28 non-specialist tertiary level hospitals. Three specialized hospitals were excluded from this analysis as they were specific for cancer and CVD. Here, NCDs represent CVD, COPD, DM and cancer where as other diseases represent other NCDs and communicable diseases (Figure 1). Out of 3,294 cases of NCDs in non-specialist institutions, majority (43%) were COPD, followed by CVD (40%), DM (12%) and cancer (5%) respectively (Figure 2).

### Comparison of different types of CVD at specialist and non-specialist institutions

Out of 1,660 CVD cases, 80% cases were diagnosed in non-specialist institutions and 20% in a specialist institution- Sahid Ganga Lal Heart Center. CVDs were classified into 7 categories based on the frequency of most occurring diseases. The main CVDs were Cerebrovascular Accident (CVA), Congestive Cardiac Failure (CCF), Ischaemic Heart Disease (IHD), Myocardial Infarction (MI), Hypertension (HTN), Rheumatic Heart Disease (RHD) and other CVDs which mainly included - hypotension, arterial fibrillation, corpulmonale etc.

**Figure 3:**
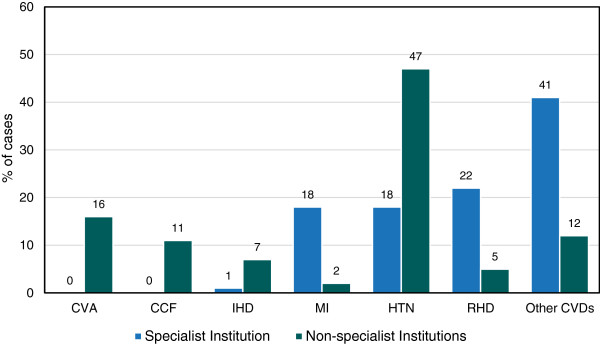
Comparison of different types of CVD at specialist and non-specialist institutions, 2009.

In non-specialist institutions, HTN comprised a large percentage (47%) of cases. However in case of specialist institution, majority (22%) of the cases were of RHD. In non-specialist institutions, CVA accounted for 16% followed by CCF (11%), whereas IHD, MI and RHD accounted for less than 10% of cases (Figure 3).

### Comparison of different types of cancer at specialist and non-specialist institutions

Out of 829 cancer cases, 18% cases were diagnosed in non-specialist institutions and 82% in specialist centers. Cancers were classified into 9 categories based on the frequency of most occurring diseases. The major cancers were of lungs, breast, cervix, ovary, oesophagus, gall bladder, rectum and stomach; while other cancers mainly included cancer of anus, appendix, lip, prostate, tongue etc.

**Figure 4:**
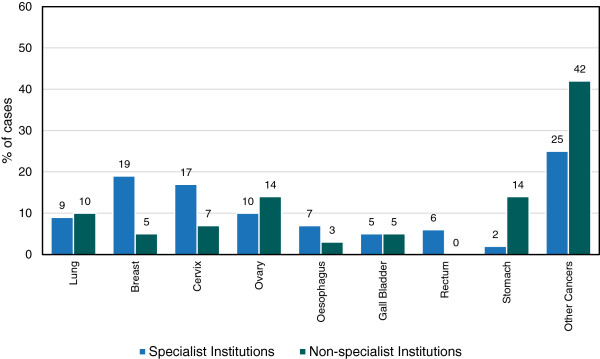
Comparison of different types of cancer at specialist and non-specialist institutions, 2009.

In non-specialist institutions, ovarian and stomach cancer accounted for majority (14%) of the cases followed by lung cancer (10%). The percentage of breast, cervix, oesophagus and gall bladder cancers were less than 10%. In contrast, in specialist centers, cases of breast cancer and cervix cancer were 19% and 17% respectively followed by ovarian cancer (10%). The percentage of lungs, oesophagus, gall bladder, stomach and rectum cancer were less than 10% (Figure 4).

### Distribution of NCDs in non-specialist institutions by age groups

The proportion of NCD cases increased with the increase in age. In older age groups (66–80 and above 80 years) approximately 52% of cases were suffering from NCDs. This figure was relatively lower in younger age groups (Table 1). In younger age group CVD was more common (49% in 35–50 years) than other NCDs. However, in older age groups COPD was more common (45% in 51–65 years, 51% in 66–80 years and 53% in above 80 years) than other NCDs. Cancer was the least common NCD in all age groups (Table 2).

**Table 1:** Distribution of NCDs in non-specialist institutions by age groups, 2009

**Age group (years)**	**NCD cases**
	**% (n)**
35-50 (n = 4768)	15.9 (761)
51-65 (n = 3750)	37.3 (1400)
66-80 (n = 1835)	51.4 (944)
> 80 (n = 358)	52.8 (189)

**Table 2:** Distribution of various NCDs in non-specialist institutions among age groups, 2009

**Age group (years)**	**CVD**	**COPD**	**Cancer**	**DM**
	**% (n)**	**% (n)**	**% (n)**	**% (n)**
35-50 (n = 761)	48.6 (370)	29.2 (222)	8.0 (61)	14.2 (108)
51-65 (n = 1400)	39.2 (549)	44.9 (628)	3.8 (53)	12.1 (170)
66-80 (n = 944)	35.7 (337)	50.8 (480)	3.2 (30)	10.3 (97)
> 80 (n = 189)	37.0 (70)	52.9 (100)	1.6 (3)	8.5 (16)

### Distribution of NCDs in non-specialist institutions by ethnic groups

Ethnicity of cases was classified into 7 groups based on the Health Management Information System, Department of Health Services, Ministry of Health and Population.^a^ The proportion of relatively advantaged janajati suffering from NCDs (42%) was higher than other ethnic groups (Table 3). CVD was most common amongst disadvantaged janajati (44%), disadvantaged non dalit terai caste group (46%) and relatively advantaged janajati (50%). On the other hand, COPD was most common amongst dalit (50%), religious minorities (53%), upper caste group (47%) and other castes (42%). Cancer was the least common NCD in all ethnic groups (Table 4).

**Table 3:** Distribution of NCDs in non-specialist institutions by ethnic groups, 2009

**Types of ethnic group**	**Total NCD**
	**% (n)**
Dalit (n = 841)	35.4 (298)
Disadvantaged janajati (n = 1877)	27.4 (514)
Disadvantaged non dalit, terai caste group (n = 683)	26.9 (184)
Religious minorities (n = 331)	26.9 (89)
Relatively advantaged janajati (n = 822)	42.1 (346)
Upper caste group (n = 4693)	31.5 (1478)
Other castes (n = 1464)	26.3 (385)

**Table 4:** Distribution of various NCDs in non-specialist institutions among ethnic groups, 2009

**Types of ethnic group**	**CVD**	**COPD**	**Cancer**	**DM**
	**% (n)**	**% (n)**	**% (n)**	**% (n)**
Dalit (n = 298)	38.6 (115)	49.6 (148)	4.4 (13)	7.4 (22)
Disadvantaged janajati (n = 514)	43.8 (225)	36.9 (190)	5.8 (30)	13.5 (69)
Disadvantaged non dalit, terai caste group (n = 184)	45.6 (84)	43.5 (80)	1.1 (2)	9.8 (18)
Religious minorities (n = 89)	32.6 (29)	52.8 (47)	4.5 (4)	10.1 (9)
Relatively advantaged janajati (n = 346)	49.7 (172)	31.2 (108)	4.9 (17)	14.2 (49)
Upper caste group (n = 1478)	37.1 (549)	47.1 (695)	4.1 (61)	11.7 (173)
Other castes (n = 385)	39.5 (152)	42.1 (162)	5.2 (20)	13.2 (51)

### Distribution of NCDs in non-specialist institutions by sex

**Figure 5:**
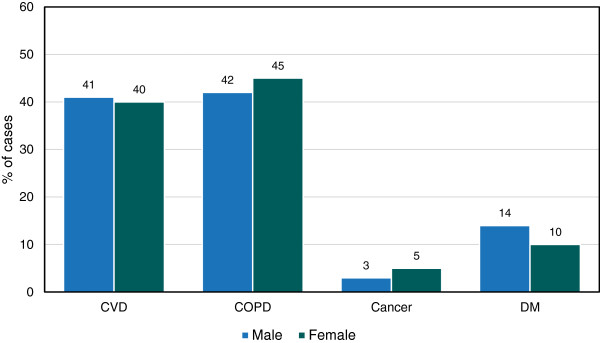
Distribution of NCDs in non-specialist institutions by sex, 2009.

In non-specialists institutions, out of all selected NCD cases (n = 3,294), 49% were females. Among males, majority (42%) were suffering from COPD followed by CVD (41%), DM (14%) and cancer (3%). Likewise among females, majority (45%) were suffering from COPD followed by CVD (40%), DM (10%) and cancer (5%). The percentage of CVD and DM was more among males than females whereas the percentage of cancer and COPD was more among females than males (Figure 5).

### Distribution of NCDs in non-specialist institutions by development regions

**Figure 6:**
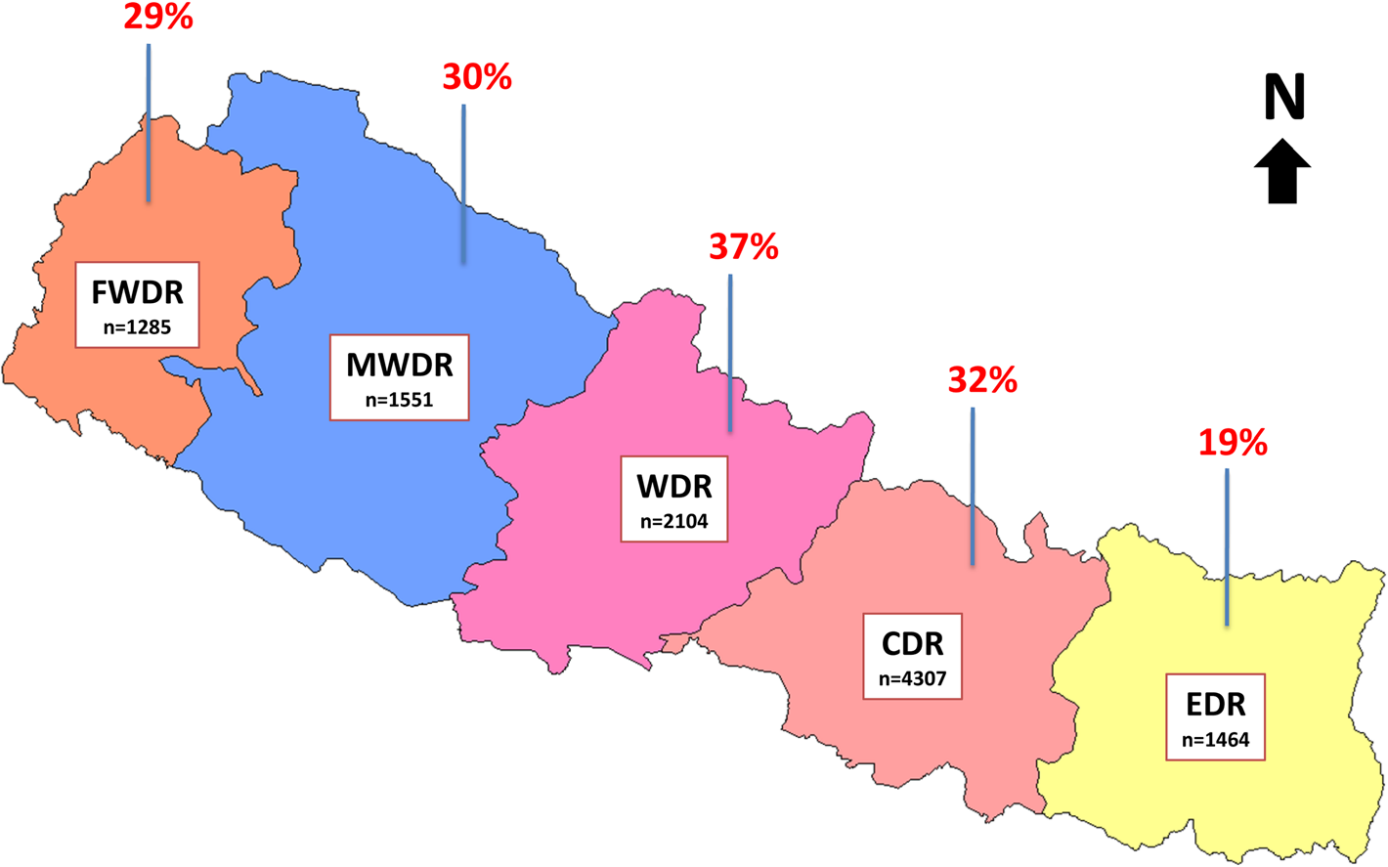
**Distribution of NCDs in non-specialist institutions by development regions, 2009.***n = number of selected cases (denominator).*

The proportion of NCDs was found to be the highest (37%) in Western Development Region (WDR), followed by Central Development Region (CDR) (32%), Mid Western Development Region (MWDR) (30%), Far Western Development Region (FWDR) (29%) and Eastern Development Region (EDR) (19%) (Figure 6). The proportion of CVD was found to be the highest in WDR (48%) and CDR (43%). Similarly, the proportion of COPD was highest in FWDR (59%), MWDR (56%) and EDR (44%). Cancer was the least common NCD in all development regions (Table 5).

**Table 5:** Distribution of various NCDs in non-specialist institutions among development regions, 2009

**Development region**	**CVD**	**COPD**	**Cancer**	**DM**
	**% (n)**	**% (n)**	**% (n)**	**% (n)**
EDR (n = 282)	32.3 (91)	43.9 (124)	10.3 (29)	13.5 (38)
CDR (n = 1383)	42.9 (594)	39.6 (547)	3.8 (52)	13.7 (190)
WDR (n = 785)	47.8 (375)	34.9 (274)	4.3 (34)	13.0 (102)
MWDR (n = 473)	31.1 (147)	56.2 (266)	5.5 (26)	7.2 (34)
FWDR (n = 371)	32.1 (119)	59.0 (219)	1.6 (6)	7.3 (27)

## Discussion

The unavailability of reliable data on NCD morbidity in many countries [2] makes it difficult to make cross country comparisons. In addition, the difference in methods applied, case definitions used and background characteristics of respondents across various studies further complicate the situation. Disease registries are a good source of information but are only available for settings where resources are abundant, rather than for entire populations. For South Asian countries, validated nationally representative estimates of cause specific mortality were not available [6]. In such a case, hospital based studies like this one probably might not lead to accurate estimates but will provide an initial baseline for further studies that estimate the burden at the population level.

In our study, the proportion of NCDs (CVD, COPD, DM and cancer) in non-specialist institutions was calculated at 31%. The inclusion of specialist health centres with higher CVD and cancer cases would have contributed to an overall increase in the percentage of NCD cases. However, we refrained from such calculations and assumptions as it might have presented a biased picture of the situation in hand. Consequently, COPD was the most common NCD followed by CVD, DM and cancer. The proportion of cancer in non-specialist institutions was lower than other selected NCDs. This might be the result of cancer patients directly visiting specialist cancer centres rather than general hospitals for treatment.

The proportion of CVD was 40% among selected NCDs in non-specialist institutions. The leading burden of CVD has also been highlighted by the WHO Global Health Observatory data (2011) for Nepal. It suggests that CVD was responsible for majority of NCD deaths, followed by cancer, COPD and DM [17]. In South East Asian Region (SEAR), chronic NCDs were responsible for 7.9 million (55%) of the estimated 14.5 million total deaths in 2008 [18]. CVDs accounted for most (45%) NCD deaths followed by chronic respiratory diseases (17%), cancers (14%) and diabetes (3%) [18].

When different types of CVD were compared between specialist and non-specialist institutions, a large proportion of CVDs (41%) in specialist institution fell in the category of other CVDs because advanced diagnostic facilities of complicated heart diseases were available only at the specialist centre. However, in non-specialist institutions there was a lack of infrastructure and diagnostic facilities for different heart diseases which might have resulted in relatively lesser (12%) percentage of other CVDs. Also there may have been referral of cases to the specialist centre. In SEAR region, the commonest CVDs were ischaemic heart disease, stroke and hypertensive heart disease [18]. However, in our study we found hypertension to be the commonest CVD in non-specialist institutions followed by cerebrovascular accident and congestive cardiac failure. Furthermore CVD was common in younger age group which follow the general trend in SEAR countries [18].

In our study a high proportion of COPD cases could be attributed to the use of traditional cooking stoves and combustion of solid biomass fuels (animal dung, crop residue, and wood) which are the main sources of indoor air pollution in Nepal [19]. The consumption of non filtered cigarettes could be another reason for the high prevalence of COPD. In mid western region of Nepal majority of patients admitted for treatment of COPD were women (60%) and the prevalence was higher in 60–69 years old (37% of overall cases) [20]. Another study from Nepal, found prevalence of 17.3% amongst patients admitted in medical ward. Prevalence was higher after the onset of middle age, with the peak at 60–69 years [21]. Eighty six percent of 1.4 million deaths due to chronic respiratory diseases in SEAR in 2008 were attributable to COPD [18].

In low-income countries lung and breast cancers were the most common diagnoses and cancers of the cervix, stomach and liver were also amongst the principle types [2]. The most common site of cancer was lungs in SEAR countries [18]. A study found that in India, cancer of the lungs and stomach were most common [22]. In our study however, cancer of the ovary, stomach, lungs and cervix were most common in non-specialist institutions. Likewise when comparing different types of cancer, the proportion of other cancers was found to be the highest as it represented the cumulative percentage of all other non-specified cancers. A hospital based study in western Nepal found cancer to be more common among males than females (1.1:1) with median age 63 and 60 years respectively [23]. For males, leading cancer sites were lung- 22.2%, larynx- 9.8% and stomach- 9% and that for females it was lung- 20%, cervix- 19.7% and breast- 7.8%. Similar findings were revealed across 7 major hospitals where cancer is diagnosed and treated [24]. On the contrary, another study observed that cancer was more common among females (56.4%) with cancer of breast (17.3%) and lungs (17.0%) as the major cancers [25].

Globally in 2008, low-income countries had the lowest (8%) prevalence of diabetes in both sexes [2]. In SEAR region, the highest prevalence of diabetes was in Bhutan (12% in males and 13% in females) and the lowest in Indonesia and Myanmar (6%–7% in both sexes) [18]. These figures are comparable to our study (12%). A lower figure could also be explained by the nature of ambulatory care of the diabetic patients resulting in less hospital visits and admissions.

Majority (66%, n = 2161) of the NCD patients in our study belonged to the age group 35–65 years. This finding has also been highlighted in global and regional reports [2,18]. It seems that the productive age groups are mostly affected which might have an indirect impact on productivity and economic growth of the country as a whole.

Here we have presented the results as obtained after implementation of the described sampling and study design to the highest standards possible. However, it might have been possible to better estimate the total number of NCD cases admitted to tertiary hospitals in Nepal at the national level. We could have estimated the total number of patients admitted to all non- specialist tertiary hospitals in Kathmandu valley by scaling up the numbers from the sample of 3 Kathmandu valley non specialist tertiary hospitals. This would require the approximate number of non-specialist tertiary hospitals in Kathmandu valley that admit NCD patients. Having obtained that, one could use the proportions from the 3 sampled and just scale the numbers up. Then, add the numbers from the 3 specialist tertiary hospitals and 25 non specialist tertiary hospitals from outside the Kathmandu valley to get the total numbers for the country.

Several limitations need to be considered when interpreting the findings of this study. First, NCD cases were identified as per the diagnosis stated in the hospital records of indoor/admitted patients only. International Classification of Diseases (ICD) codes or any other standardized case definitions were not used. Second, information on different types of COPD and diabetes (type 1 or type 2) was not collected as they were not available. Third, the study did not take in to account the issues related to multiple hospital admissions or multiple diagnosis of a single patient. Despite these limitations, this nationwide hospital based study is a first of its kind in Nepal and is a good representation of the hospital based NCD burden. We are convinced that this study will serve as a much needed baseline for future studies to build upon.

During the course of this study we also observed that the recording system in most of the hospitals needs to be improved and standardized. A uniform format to record the patient’s details including diagnosis should be devised and implemented. This would lead to better reporting of morbidity and more accurate estimates of burden at the national level. It is also advisable to consider updating into standardized computer based electronic systems with centralized database which would help maintain the data at various levels of the health system.

## Conclusions

This cross sectional study was carried out to estimate the hospital based prevalence of NCDs. It was able to reveal the evidence of NCD problem that Nepal is facing. The study found that 31% of the cases diagnosed in non-specialist institutions in 2009 were one of the four major NCDs (CVD, COPD, DM and cancer). Ovarian, stomach and lung cancer were the main cancers accounting for 38% of distribution. Majority of the CVD cases were of hypertension (47%) followed by cerebrovascular accident, congestive cardiac failure, ischemic heart disease, rheumatic heart disease and myocardial infarction. These findings reflect that Nepal is also facing the surging burden of NCDs similar to other developing nations in South East Asia.

In conclusion, the prevalence of NCD is substantial in Nepal and should be regarded as a public health problem. Although evidence suggesting pandemic of NCDs is irrefutable, as also seen in this study, there is a paucity of program to detect, manage and prevent these diseases in Nepal. The government, non-government and community based organizations are still fighting to tackle the burden of infectious diseases. Unless urgent and specific focus on preventing, treating and controlling NCDs are targeted, the burden of NCDs will soon be unbearable to a poor nation like Nepal. This study has provided a background data on NCDs in Nepal which should prove useful for the concerned organizations to focus and contribute towards the prevention, control and reduction of NCD burden and its risk factors.

## Endnotes

^a^Ethnic codes as defined by the Health Management Information System:

Dalits: hills of Kami, Damai, Sharki, Gaine, Badi; Disadvantaged Janajati: hills of Magar, Tamang, Rai, Limbu, Sherpa, Bhote, Walung, Sunuwar, Kumal, Jirel, Danuwar, Thami, Raji; Disadvantaged non Dalit Terai caste groups: Yadav, Teli; Religious minorities: Muslims, Chureto; Relatively advantaged Janajatis: Newar, Thakali, Gurung; Upper caste groups: Brahmin, Chhetri, Thakuri, Sanyashi, Raajput, Kayastha, Baniya, Marwadi, Jaire, Nurang, Bengali.

## Competing interests

The authors declare that they have no competing interests.

## Authors’ contributions

GPB participated in the design of the study, supervised the study, and participated in analysis and preparation of the manuscript. MRA drafted the manuscript, performed statistical analysis and made critical revisions to the paper. MD, SN and CB were involved in design, supervision and analysis of the study. All authors read and approved the final manuscript.

## Pre-publication history

The pre-publication history for this paper can be accessed here:

http://www.biomedcentral.com/1471-2458/14/23/prepub
